# Where does the time go? Temporal patterns of pumping behaviors in mothers of very preterm infants vary by sociodemographic and clinical factors

**DOI:** 10.3389/fnut.2024.1278818

**Published:** 2024-01-30

**Authors:** Aloka L. Patel, Amelia Tan, Amelia Bucek, Judy Janes, Katie McGee, Delaney Mulcahy, Paula Meier, Tricia J. Johnson

**Affiliations:** ^1^Department of Pediatrics, Rush University Medical Center, Chicago, IL, United States; ^2^Northwestern University, Chicago, IL, United States; ^3^Department of Health Systems Management, Rush University, Chicago, IL, United States

**Keywords:** preterm infants, milk expression, health disparities, lactation, breast milk, mother’s own milk, neonatal intensive care unit

## Abstract

**Background:**

Mothers of very preterm (<32 weeks gestational age [GA]) infants are breast pump dependent and have shorter duration of milk provision than mothers of term infants. The opportunity (i.e., time) cost of pumping and transporting mother’s own milk (MOM) from home to the NICU may be a barrier. There is a paucity of data regarding how much time mothers actually spend pumping.

**Objective:**

To investigate the variation in pumping behavior by postpartum week, maternal characteristics, and infant GA.

**Methods:**

Prospectively collected pump log data from mothers enrolled in ReDiMOM (Reducing Disparity in Mother’s Own Milk) randomized, controlled trial included pumping date and start time and end time of each pumping session for the first 10 weeks postpartum or until the infant was discharged from the NICU, whichever occurred first. Outcomes included number of daily pumping sessions, number of minutes spent pumping per day, and pumping behaviors during 24-h periods, aggregated to the postpartum week. Medians (interquartile ranges) were used to describe outcomes overall, and by maternal characteristics and infant GA.

**Results:**

Data included 13,994 pump sessions from 75 mothers. Maternal characteristics included 55% Black, 35% Hispanic, and 11% White and 44% <30 years old. The majority (56%) of infants were born at GA 28–31 weeks. Mothers pumped an average of less than 4 times per day, peaking in postpartum week 2. After accounting for mothers who stopped pumping, there was a gradual decrease in daily pumping minutes between postpartum weeks 2 (89 min) and 10 (46 min). Black mothers pumped fewer times daily than non-Black mothers after the first 2 weeks postpartum.

**Conclusion:**

On average mothers pumped less intensively than the minimum recommendation of 8 times and 100 min per day. However, these pumping behaviors represent significant maternal opportunity costs that should be valued by the institution and society at large.

## 1 Introduction

It is well established that mother’s own milk (MOM) feedings from the infant’s mother reduce the risk of prematurity related complications during and after the neonatal intensive care unit (NICU) hospitalization, including infections, rehospitalizations, and neurodevelopmental delay in very preterm (VPT, birth gestational age < 32 weeks) and very low birth weight (VLBW, birth weight < 1,500 g) infants (1–18). This risk reduction is attributed to the unique nutritional, immunomodulatory, anti-inflammatory, and epigenetic components of MOM that protect and stimulate the development of many body organs, enzymatic and metabolic pathways, and hormonal responses in the early post-birth period (19–31). Selected specific effects and components include: growth and development of the brain, gut, lungs, programming of growth, tolerance to antigens and induction of selective enzymes via complex complementary mechanisms such as the MOM/infant gut microbiome, oligosaccharides, and MOM-borne lipases, amylases, proteases, adipokines, extracellular vesicles and micro RNAs. These protective functions are especially important for the smallest, least mature infants due to their immunocompromised state. Furthermore, several studies reveal a dose–response relationship between the amount (dose) and duration (exposure period) of human milk (HM) received by the infant and the degree of protection from these acute and chronic illnesses as well as their short- and long-term costs (1, 8–12, 14, 15, 17, 18, 32).

In the US, VPT infants born to non-Hispanic Black (Black) mothers are significantly less likely to receive any MOM at time of NICU discharge when compared to non-Hispanic White (White) and Hispanic mothers (33–36). This disparity in duration of MOM provision directly impacts the ability of Black infants to receive MOM for the recommended duration to garner the many long-term health benefits associated with exclusive MOM feedings for the first 6 months of life and continued MOM feedings through 2 years of age (7, 37). These differences translate into a lifetime of health and economic advantages for MOM-fed versus formula-fed infants and their families.

Mothers of VPT infants encounter many challenges in providing MOM compared to mothers of healthy, term infants. These include preterm delivery, maternal illnesses such as preeclampsia, increased rates of cesarean delivery, stress of the NICU hospitalization, separation from their infant, and reliance on a breast pump to initiate and sustain MOM provision (38). Several studies have demonstrated the importance of pumping behaviors (e.g., daily minutes spent pumping, daily number of pumping sessions, inter-pump intervals) on establishing and sustaining MOM provision during the NICU hospitalization, including initiating milk expression within the first 6 h after birth (39–42), pumping during the early morning (43), and adequate pumping frequency, especially in the first 4–14 days postpartum (35, 39–45). Stimulation of the mammary gland by the infant or breast pump during the transition from secretory differentiation to secretory activation is critical, with programming effects on lactocytes (38, 39, 46). Recommendations for pump-dependent women include pumping 8–12 times daily, especially in the first 14 days postpartum (47, 48). Furthermore, studies suggest a minimum of 5–6 pumping sessions per day during the first 1–3 weeks postpartum is associated with establishing appropriate MOM volume, called coming to volume (CTV, pumping ≥500 mL/day MOM) by 14 days postpartum, or continued MOM provision to NICU discharge (35, 40, 41, 43, 49). A study of 415 mothers of VPT infants from 2008 to 2012 indicated that CTV was the most significant predictor of MOM provision at NICU discharge (odds ratio [OR] 7.46, 95% confidence interval [CI] 3.26 to 17.07, *p* < 0.001) (35). Furthermore, this study showed that differences in pumping frequency during the first 14 days postpartum mediated the Black-White (indirect effect: b = 0.40, 95% CI 0.26 to 0.57) and Black-Hispanic (indirect effect: b = 0.18, 95% CI 0.08 to 0.31) disparity in MOM feeding at NICU discharge with White and Hispanic mothers pumping more frequently than Black mothers. White mothers pumped an average of 5.2 times/day (standard deviation [sd] = 1.2), Hispanic mothers pumped 4.5 times/day (sd = 1.3) and Black mothers pumped 4.0 times/day (sd = 1.2) in the first 14 days (35).

To date, few studies have examined how frequently and when mothers of VPT infants actually pump and how pumping behaviors vary by maternal and infant characteristics (35, 39–41, 43, 49, 50). The aims of this study were to (1) examine the variation in pumping behaviors (i.e., average daily time spent pumping, average daily number of pumping sessions, percent of days with an early morning pumping session [between 1:00 a.m. and 4:59 a.m. (43)], and percent of days with at least 5 pumping sessions) by postpartum week, and (2) provide a descriptive comparison of pumping behaviors by maternal and infant characteristics, including maternal race/ethnicity, age at birth, and primary payment source and infant gestational age (GA).

## 2 Materials and methods

### 2.1 Study population

Data for this study were obtained from the ReDiMOM (**Re**ducing **Di**sparity in Receipt of **M**other’s **O**wn **M**ilk in Very Low Birth Weight Infants) randomized controlled trial (RCT) at Rush University Medical Center enrolling adult mothers and their inborn VPT infants. Briefly, maternal inclusion criteria for the ReDiMOM RCT included age ≥ 18 years, fluent in English or Spanish, willing and able to share a valid Social Security number and planned to provide at least some MOM feedings. Infant inclusion criteria were < 32 0/7 weeks GA at birth, no significant congenital anomalies or chromosomal defects, and ≤ 144 h (6 days) of age at the time of enrollment. Therefore, mothers who planned to provide MOM were included in the analysis, regardless of the actual proportion of feedings that consisted of MOM. ReDiMOM tests the effectiveness and cost-effectiveness of a three-part intervention bundle designed to offset opportunity (i.e., time) and out-of-pocket costs of MOM provision, including a hospital-grade breast pump for use at home, transportation of pumped MOM from home to the NICU, and payment for time spent pumping (51). This study included pumping records for the first 75 mothers who completed study activities and documented at least one pumping session in the pumping log. Data included in this analysis were limited to pumping records for the first 10 weeks of life (WOL) or the infant’s discharge from the NICU, whichever occurred first.

### 2.2 Measures

Mothers recorded each pumping session either on a paper log or in an electronic REDCap log, including the start and stop date and time of each pumping session. For paper logs, mothers either returned them to study staff or photographed the logs and uploaded the files to a secure REDCap questionnaire. Study staff manually entered paper log data into an Excel spreadsheet and uploaded the spreadsheet into the study’s REDCap database.

Duration of each pumping session was calculated in minutes by subtracting the start date and time from the end date and time. Pumping sessions that were missing the start time, end time or were for 0 min in duration (*n* = 216), greater than 75 min (*n* = 138), less than 0 min (*n* = 153), or included pumping dates prior to the infant’s birth date (*n* = 10) were excluded, for a total of 3.6% of pumping records dropped from the analysis. Days without any pumping records that were after the first date of pumping but before discharge were assumed to have 0 min spent pumping and 0 pumping sessions. Overall, 33% of the subjects had pumping data for every day of their infant’s NICU stay, after the first date of pumping. An additional 20% of subjects had ≤20% of NICU days with no pumping sessions, and 11% had 21–40% of NICU days with no pumping sessions (Supplementary Figure S1). A sensitivity analysis was conducted by excluding subjects from the calculations after they had discontinued pumping (last recorded pumping date), leaving only those subjects who were still pumping based on the pumping logs in the analysis.

Outcomes included average daily number of pumping sessions (i.e., frequency) by WOL, average daily time spent pumping (i.e., duration) by WOL, and other pumping behaviors, including the percent of days with at least 5 pumping sessions by WOL and percent of days with an early morning pumping session by WOL (43). For each WOL, average daily number of pumping sessions per week was calculated as $\overset{n}{\underset{i=1}{\sum }}\mathrm{number}\;\mathrm{of}\;\mathrm{pumping}\;\mathrm{session}s_{i}/n\text{.}$ Average daily time spent pumping was calculated as $\overset{n}{\underset{i=1}{\sum }}\mathrm{daily}\;\min_{i}/n$ for each week where *i* = day and *n* = number of days in a week. The first week of pumping and the last week before discharge may have had fewer than 7 days of data. For the sensitivity analysis that included pumping records from the first to last date of pumping, the last week of pumping may have had fewer than 7 days of data. To calculate the proportion of days with at least 5 pumping sessions in each WOL, we classified each day as either having at least 5 pumping sessions or not and divided by the number of days in the week. To calculate the proportion of days with an early morning pumping session in each WOL, we classified each day by whether the mother had a pumping session that started between 1:00 a.m. and 4:59 a.m. (43) and divided the number of days in the week with an early morning pumping session by the total number of days in the week. The percent of days with at least 5 pumping sessions and percent of days with an early morning pumping session were calculated only for those subjects who were still pumping at each WOL (“while pumping”), excluding subjects that had discontinued pumping. Results are reported as medians of the average for each outcome.

Independent variables included maternal self-identified race/ethnicity (Black, Hispanic, White), age (<30 years versus ≥30 years), and primary payment source (Medicaid versus private insurance) and infant GA at birth (<28 weeks versus 28–31 weeks). Other variables included to describe the sample were delivery mode (vaginal versus cesarean delivery), 5-min Apgar Score, number of prior deliveries (none, 1, 2, 3 or more), whether the subject provided any MOM to previous children, and maternal goal for MOM provision at NICU discharge (reported at the time of study enrollment).

### 2.3 Statistical analysis

Frequency distributions and medians (interquartile range [IQR]) were used to describe the sample and pumping behaviors by WOL. Heatmaps and line graphs were used to graphically display outcomes by WOL. Line graphs included four panels: (A) median of the average number of daily pumping sessions by WOL, (B) median of the average number of daily pumping minutes by WOL, (C) median of the average percent of days each week with at least 5 pumping sessions by subjects who continued to pump MOM by WOL (i.e., while pumping), and (D) median of the average percent of days each week with at least 1 early morning pumping session by subjects who continued to pump MOM by WOL (i.e., while pumping). Line graphs were created for all mothers and stratified by maternal race/ethnicity, maternal payment source, maternal age, and infant GA.

## 3 Results

### 3.1 Description of the sample

The sample included 75 mothers and 13,994 recorded pumping sessions, with 55% of the mothers Black, 35% Hispanic and 11% White (Table 1). There was no significant difference in the distribution of mothers by race/ethnicity and ReDiMOM study arm (*p* = 0.870), with 56% of Black, 50% of Hispanic, and 50% of White mothers randomized to the intervention arm. Overall, 44% were < 30 years, 55% were insured by Medicaid, and 56% delivered an infant at 28–31 weeks GA. The average number of pumping sessions/day and pumping minutes/day peaked in WOL 2, with a median of 3.9 pumping sessions/day (IQR 1.7–5.6) and 89 pumping minutes/day (IQR 36–122), decreasing to 2.0 pumping sessions (IQR 0–4.8) and 46 pumping minutes/day (IQR 0–98) in WOL 10 (Table 2). The percentage of days with at least 5 pumping sessions peaked in WOL 6 and 7, with a median of 57.1% (IQR 0–100.0%), partially due to mothers with low pumping frequency discontinuing pumping and being dropped from the analysis. The average percent of days with an early morning pumping session was highest in WOL 3 and 6, with a median of 29%.

**Table 1:** Description of the sample, *N* = 75.

Characteristic	
Maternal race/ethnicity, *n* (%)	
Black	41 (54.7)
Hispanic	26 (34.7)
White	8 (10.7)
Maternal age, *n* (%)	
<30 years	33 (44.0)
30 years and older	42 (56.0)
Primary payment source, *n* (%)	
Commercial	34 (45.3)
Medicaid	41 (54.7)
Infant gestational age at birth, *n* (%)	
<28 weeks	33 (44.0)
28–31 weeks	42 (56.0)
Delivery mode, *n* (%)	
Vaginal	17 (22.7)
Cesarean delivery	58 (77.3)
5-Minute Apgar Score, median (IQR)	8 (6, 8)
Prior deliveries *n* (%)	
None	38 (50.7)
1	13 (17.3)
2	16 (21.3)
3 or more	8 (10.7)
Provided MOM to previous child(ren), *n* (%)*	28 (75.7)
Goal to provide exclusive MOM at NICU discharge, *n* (%)**	62 (83.8)

IQR, interquartile range (25th percentile, 75th percentile). IQR, interquartile range (25th percentile to 75th percentile), MOM, mother’s own milk, NICU, neonatal intensive care unit. *Denominator is subjects with other children (*n* = 37); **n = 74.

**Table 2:** Maternal pumping patterns by week of life.

Week of Life	During NICU stay*			While pumping**		
	*N*	Average daily number of pumping sessions per mother median (IQR)	Average daily minutes per mother median (IQR)	*N*	Percent of days with at least 5 pumping sessions per mother median (IQR)	Percent of days with early morning pumping session per mother median (IQR)
1	70	3.6 (1.7, 4.8)	69 (36, 108)	70	31.0 (0, 75.0)	16.7 (0, 40.0)
2	74	3.9 (1.7, 5.6)	89 (36, 122)	68	35.7 (0, 85.7)	14.3 (0, 42.9)
3	75	3.7 (0.6, 6.0)	80 (13, 131)	65	42.9 (0, 100.0)	28.6 (0, 57.1)
4	74	3.4 (0.1, 5.7)	71 (3, 128)	60	42.9 (0, 100.0)	14.3 (0, 57.1)
5	74	3.1 (0, 5.6)	62 (0, 119)	54	35.7 (0, 100.0)	14.3 (0, 42.9)
6	72	2.8 (0, 5.4)	57 (0, 106)	49	57.1 (0, 100.0)	28.6 (0, 71.4)
7	67	2.4 (0, 5.5)	54 (0, 108)	43	57.1 (0, 100.0)	14.3 (0, 57.1)
8	59	2.3 (0, 5.0)	56 (0, 103)	36	42.9 (7.1, 100.0)	14.3 (0, 35.7)
9	54	2.0 (0, 5.4)	48 (0, 102)	34	35.7 (0, 100.0)	14.3 (0, 42.9)
10	49	2 (0, 4.8)	46 (0, 98)	29	33.3 (0, 100.0)	25.0 (0, 42.9)

IQR, interquartile range (25th, 75th percentiles). * *N* by week of life varies across the 10 weeks, due to variation in the first day with pumping data reported and infants discharged from the NICU in the first 10 weeks of life. Reported values include subjects that had stopped pumping, with 0 min entered for their data. ** *N* by week of life varies across the 10 weeks, due to variation in the first day with pumping data reported, subjects stopping pumping during the NICU stay, and infants discharged from the NICU in the first 10 weeks of life.

### 3.2 Overall pumping sessions, pumping duration and other pumping behaviors

**Figure 1:**
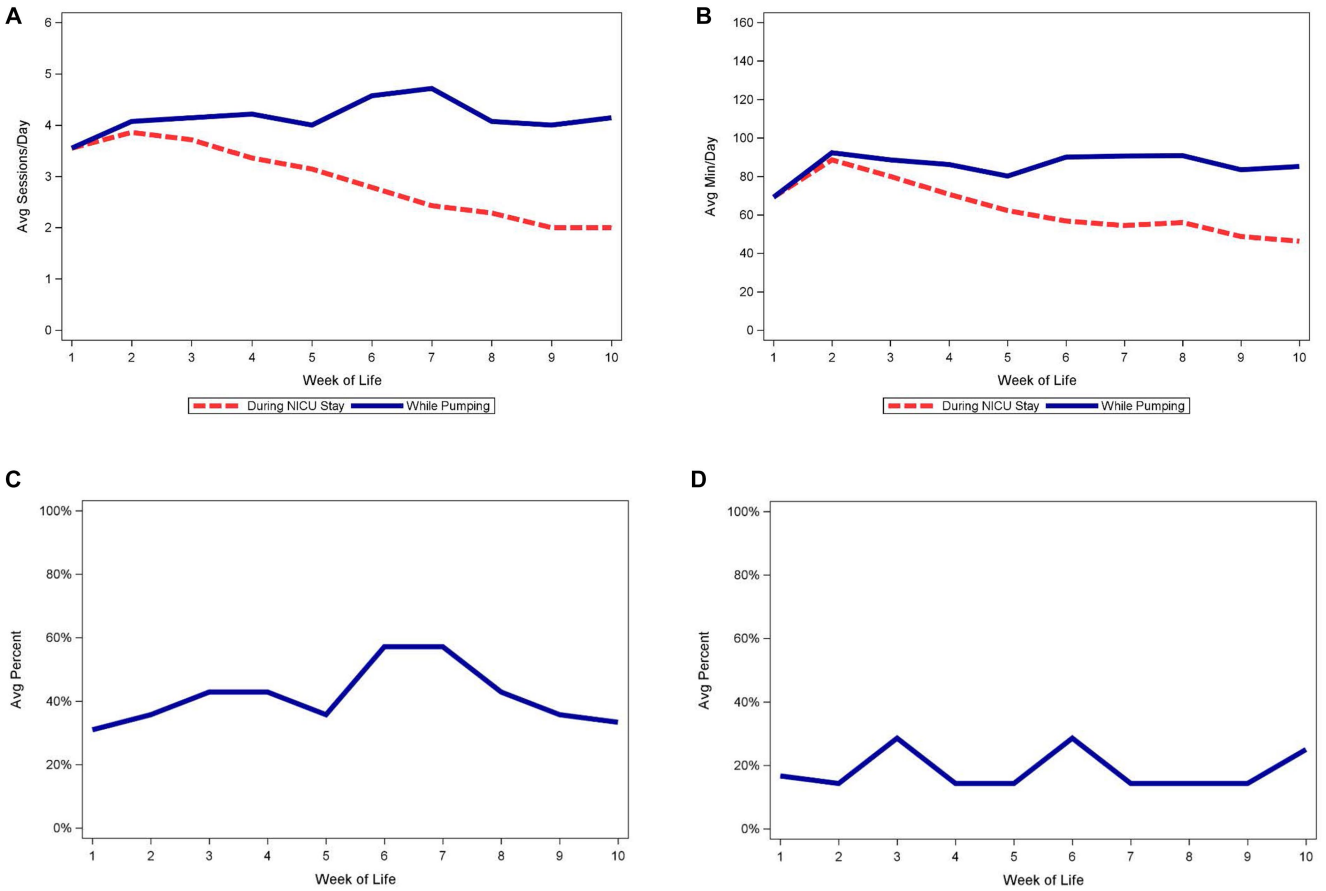
Median of average daily pumping sessions and pumping minutes by week of life. **(A)** Median of average daily pumping sessions. **(B)** Median of average daily pumping minutes. **(C)** Median of average percent of days with at least 5 pumping sessions, while pumping. **(D)** Median of average percent of days with at least 1 early morning pumping session, while pumping.

Figure 1 demonstrates the medians of the average number of daily pumping sessions, average daily pumping durations, and other pumping behaviors by WOL. Panels A and B demonstrate the difference in the number of daily pumping sessions and duration based on utilizing data from the entire sample versus only subjects that continued to pump. A notable but not unexpected difference is evident, with relatively constant frequency and duration noted after week 2 when limiting the data to subjects who continued pumping compared to the steady decline noted when subjects who had discontinued pumping were also included in the data. Panels C and D display pumping patterns for subjects that continued to pump. Using data from subjects who continued to pump, we found a minority of subjects pumped at least 5 times per day in the first 5 weeks post-partum and a very small minority had at least 1 early morning pumping session.

**Figure 2:**
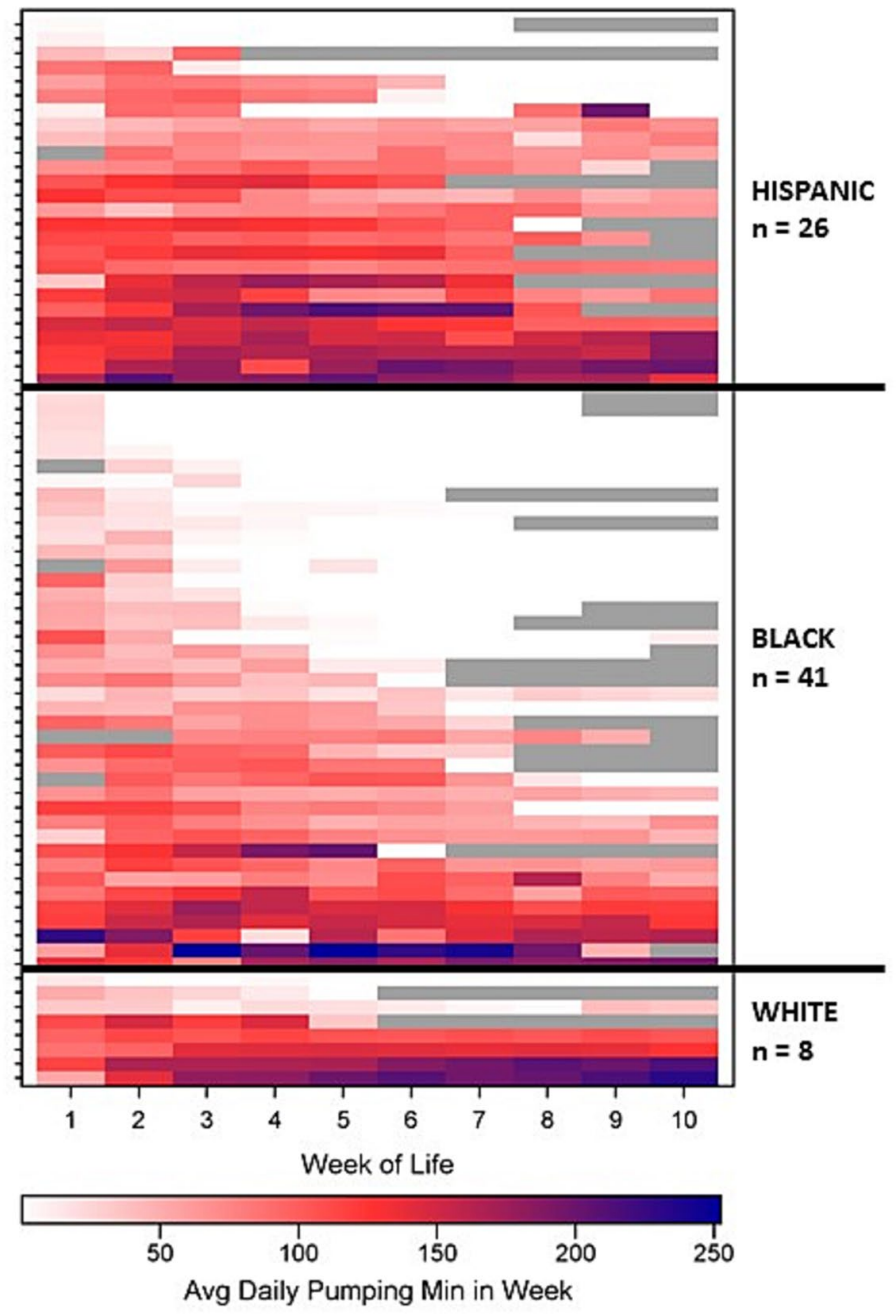
Average daily pumping minutes by week of life grouped by maternal race/ethnicity. Gray shading indicates weeks after discharge.

Figure 2 displays a heatmap of the intensity of average daily pumping minutes by WOL by maternal race/ethnicity for all subjects, illustrating both inter-subject (i.e., between mother) and intra-subject (i.e., within mother, over time) variation in minutes/day across time. Figure 2 qualitatively shows greater inter-subject and intra-subject variation in pumping duration for Black mothers compared to Hispanic and White mothers. Supplementary Figure S2 includes heatmaps for the intensity of average number of daily pumping sessions, average daily pumping durations, and pumping patterns by WOL, illustrating greater inter-subject than intra-subject variation in all outcomes (Supplementary Table S1).

### 3.3 Pumping sessions, pumping duration and other pumping behaviors by maternal and infant characteristics

**Figure 3:**
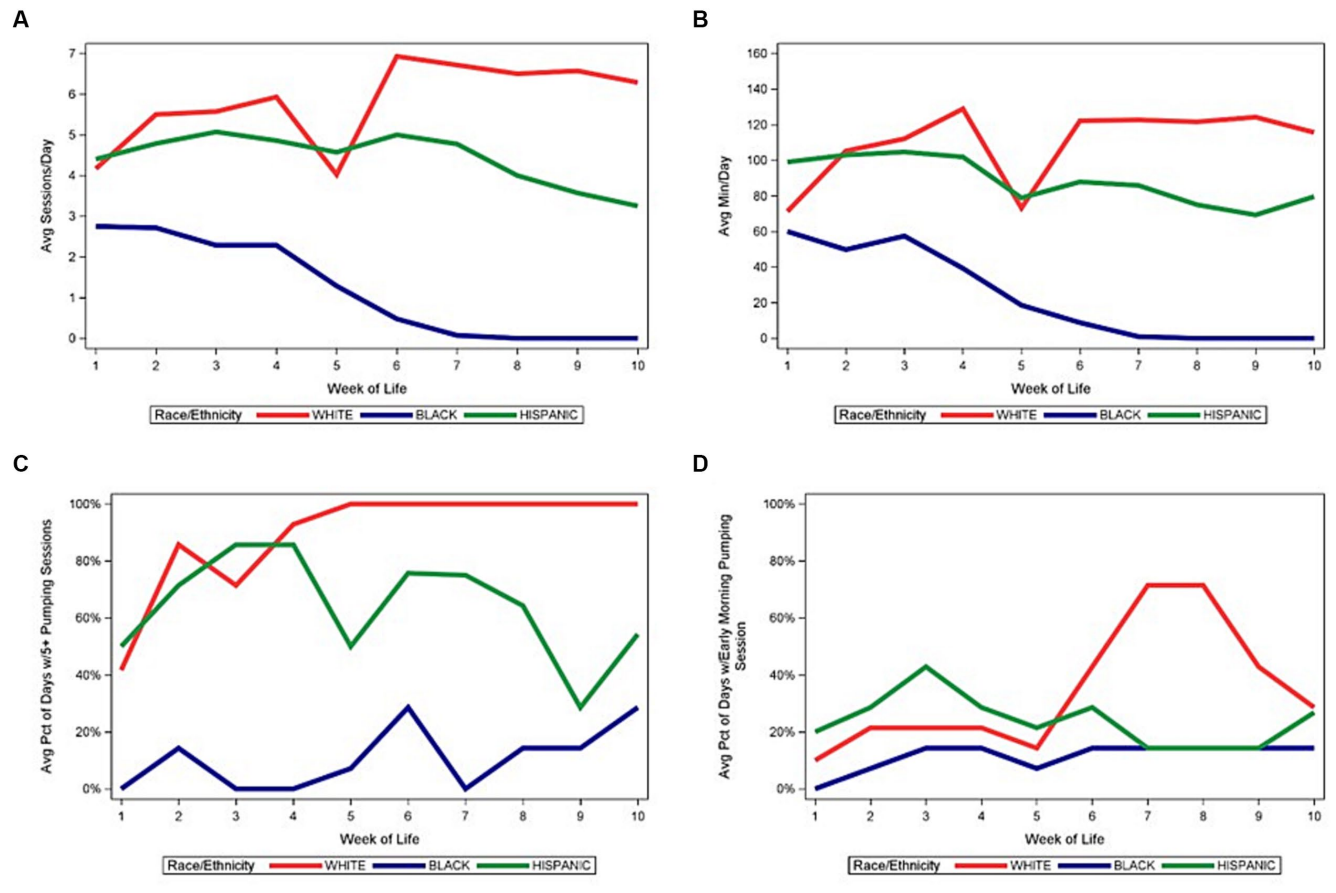
Median of pumping patterns by maternal race/ethnicity and week of life during NICU stay. **(A)** Median of average daily pumping sessions. **(B)** Median of average daily pumping minutes. **(C)** Median of average percent of days with at least 5 pumping sessions, while pumping. **(D)** Median of average percent of days with at least 1 early morning pumping session, while pumping.

Figure 3 illustrates the medians of the average number of daily pumping sessions and average daily pumping durations (for the NICU stay), and other pumping behaviors by WOL and by maternal race/ethnicity for the entire sample. The average number of daily pumping sessions and pumping duration show similar patterns (panels A and B). The average number of daily pumping sessions increases between WOL 1 and 6 for White mothers and then remains relatively constant until WOL 10, except for a transient decline at WOL 5 (panel A). For Hispanic mothers, average daily pumping sessions remain relatively constant between WOL 1–6 and then decrease through WOL 10. For Black mothers, average number of daily pumping sessions remains relatively constant between WOL 1–3 and then decreases drastically to WOL 6, after which the median is 0 or discontinued pumping.

**Figure 4:**
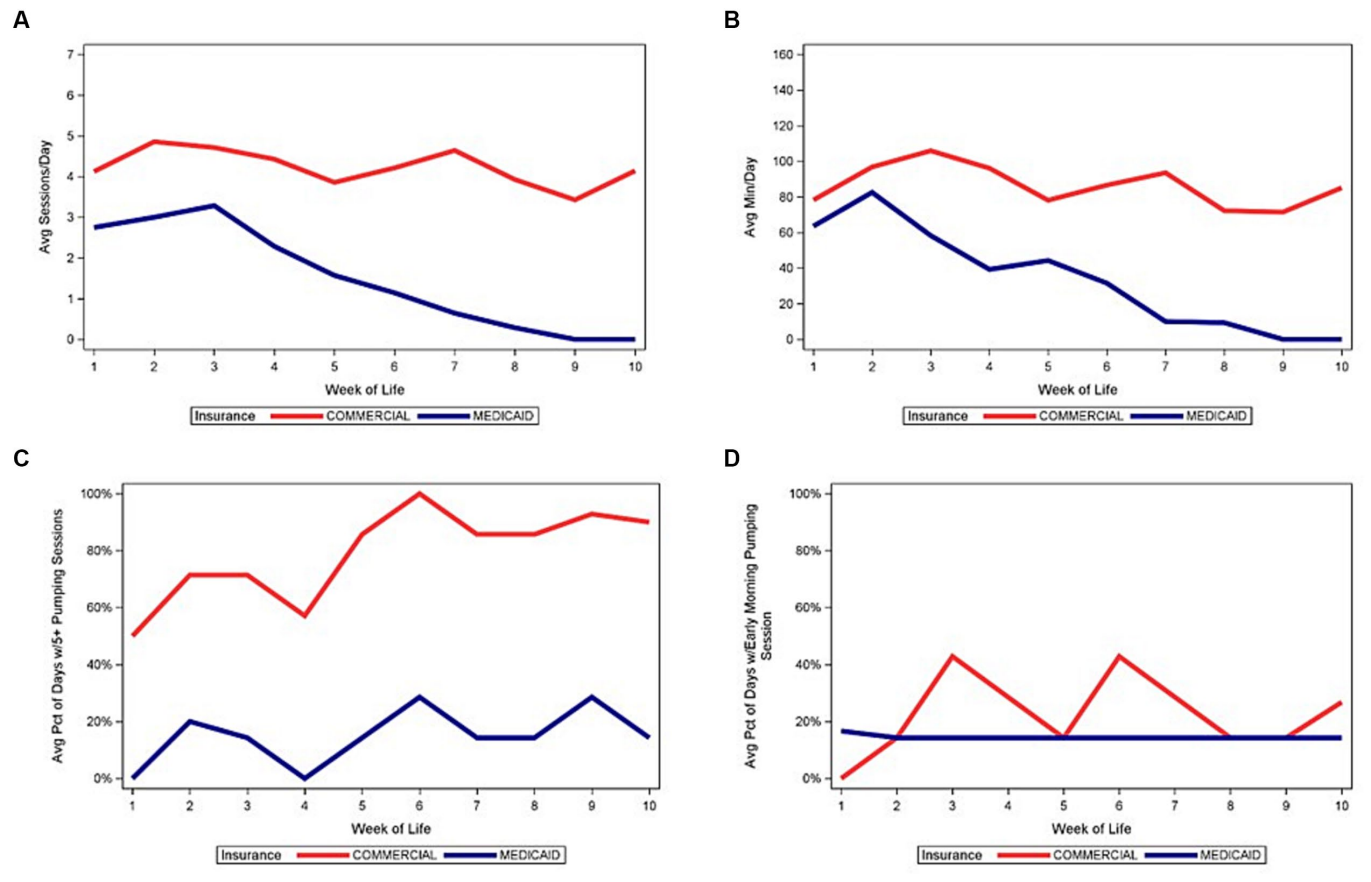
Median of pumping patterns by maternal insurance and week of life during NICU stay. **(A)** Median of average daily pumping sessions. **(B)** Median of average daily pumping minutes. **(C)** Median of average percent of days with at least 5 pumping sessions, while pumping. **(D)** Median of average percent of days with at least 1 early morning pumping session, while pumping.

**Figure 5:**
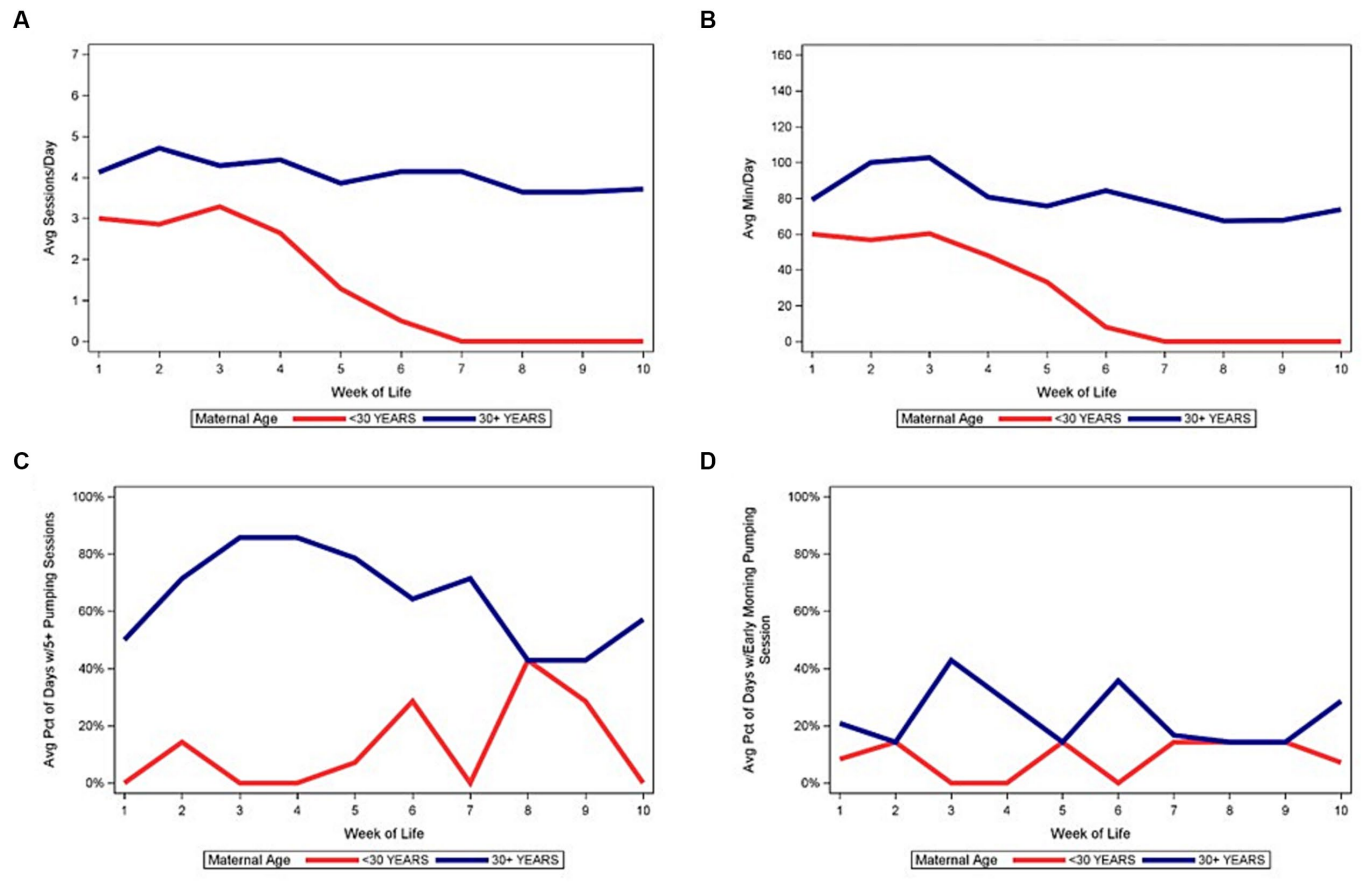
Median of pumping patterns by maternal age and week of life during NICU stay. **(A)** Median of average daily pumping sessions. **(B)** Median of average daily pumping minutes. **(C)** Median of average percent of days with at least 5 pumping sessions, while pumping. **(D)** Median of average percent of days with at least 1 early morning pumping session, while pumping.

**Figure 6:**
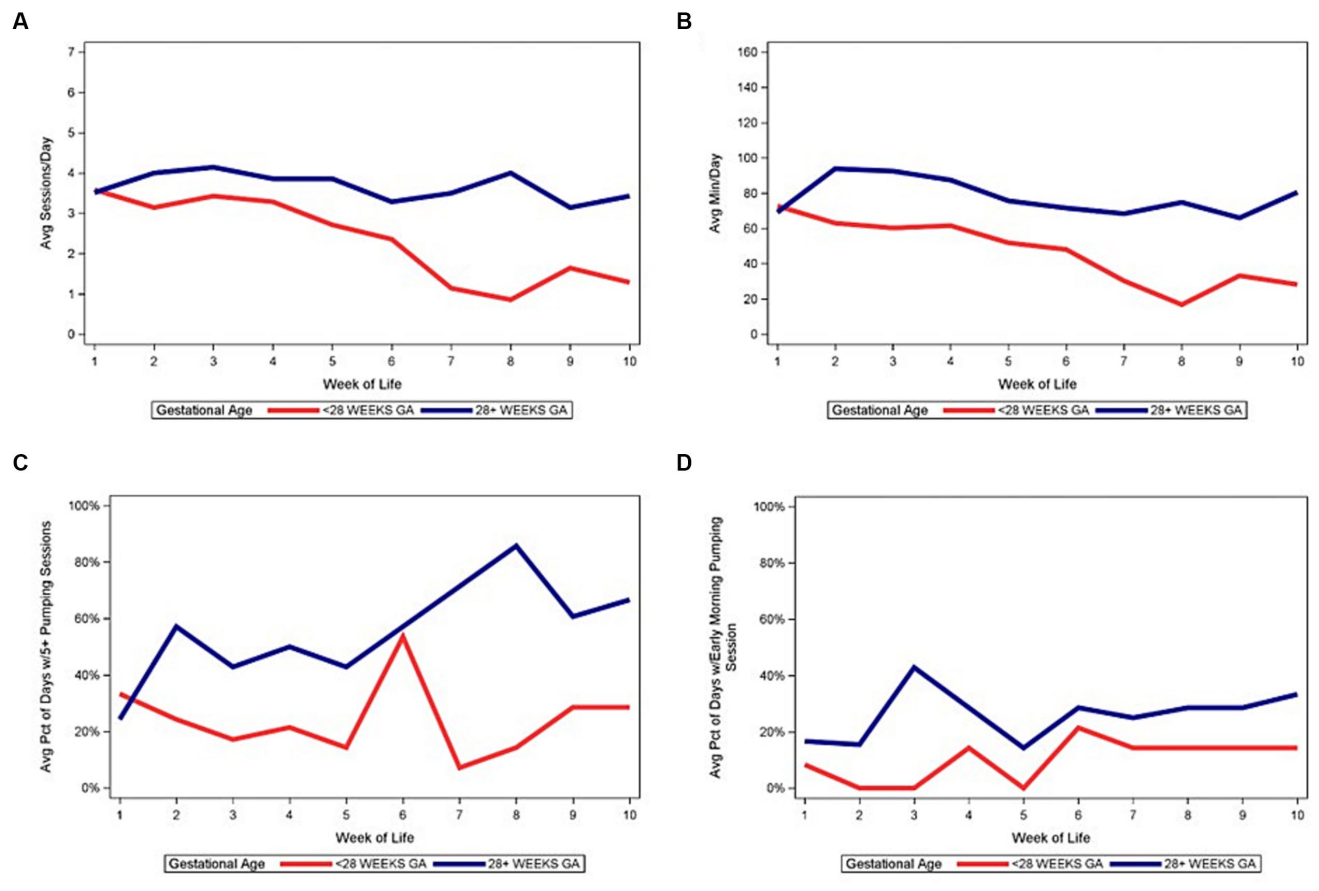
Median of pumping patterns by infant gestational age and week of life during NICU stay. **(A)** Median of average daily pumping sessions. **(B)** Median of average daily pumping minutes. **(C)** Median of average percent of days with at least 5 pumping sessions, while pumping. **(D)** Median of average percent of days with at least 1 early morning pumping session, while pumping.

Figures 4–6 depict the medians of the average number of daily pumping sessions, average daily pumping durations and pumping patterns by WOL and by payment source, maternal age, and infant GA. The average number of daily pumping sessions remain consistently higher for mothers with commercial insurance compared to those with Medicaid (Figure 4A), consistently higher for mothers aged 30 and older compared to mothers under age 30 (Figure 5A), and modestly higher for infants born 28–31 weeks GA compared to those with infants born <28 weeks GA (Figure 6A).

## 4 Discussion

For mothers of VPT infants, pumping frequency in the early weeks after delivery is positively correlated with later lactation outcomes, including achievement of CTV by postpartum day 14 (38, 43, 45), duration of MOM provision (35, 40, 51), receipt of MOM at NICU discharge and exclusive MOM provision in the NICU (42). Studies examining pumping behavior and lactation outcomes have inconsistently included race/ethnicity in analyses (35, 40, 43) despite disparities in MOM provision (33–36). Although these studies have demonstrated a disparity between White mothers and other mothers in achievement of CTV, or in the receipt of MOM at specific time points, such as postpartum day 14 or NICU discharge, no previous study has described detailed pumping behaviors for mothers of VPT infants, and whether or how these behaviors vary by maternal race/ethnicity and other demographic characteristics.

Our data demonstrate racial and ethnic differences in pumping behaviors, with Black mothers demonstrating shorter and less intensive pumping compared to White and Hispanic mothers. Although our previous research has demonstrated similar rates of MOM provision at NICU discharge by White and Hispanic mothers (35), the current data demonstrate that the pumping behavior between White and Hispanic mothers are initially similar but then diverge around 4–6 weeks postpartum. Mothers in this study were enrolled in the ReDiMOM trial, for which randomization was stratified by maternal race/ethnicity and infant GA (52). Therefore, despite the economic intervention available to the intervention arm, the three racial/ethnic groups were similarly distributed between the control and intervention study arms. We also noted differences associated with primary payment source, with greater pumping frequency and duration in commercially insured mothers. During the first 2 WOL, publicly and privately insured mothers pumped a similar number of times each day, but during WOL 2–10, privately insured mothers pumped significantly more frequently.

Intra-individual differences were also evident and widely variable, with the most common pattern consisting of a gradual decline in pumping frequency and duration with prolonged NICU hospitalization. A small minority of mothers were able to maintain a consistent pattern of pumping duration, and they were more likely to be White, aged 30 years or older, or those with commercial insurance. This gradual decline in pumping intensity is commonly seen in mothers of VPT due to challenges associated with prolonged pump-dependency. Researchers have repeatedly demonstrated the vital importance of frequent pumping in the first 1–2 weeks postpartum to program the mammary gland for later milk production, with animal studies demonstrating changes in gene expression and cellular and hormonal changes associated with milk expression frequency (35, 39, 42, 43, 45, 51, 53–56). In the early postpartum period, reductions in pumping frequency can negatively impact milk, with short-term changes in pumping frequency associated with changes in milk biomarkers. This lack of stability in mammary function during this critical early period heightens the importance of understanding pumping behaviors to develop actionable interventions.

Standard lactation recommendations given to mothers of VPT infants include pumping 8–12 times/day in the early postpartum period to mimic a healthy term newborn (47, 48, 57), although the actual minimum pumping frequency requirement is unknown (7). A recent study by Mago-Shah et al. suggested that pumping at least 5 times per day by day 5 and pumping once in the early morning were independently associated with CTV (43), which is in turn associated with continued MOM provision at NICU discharge (35, 58). This is aligned with findings from an observational study of mothers of infants <34 weeks GA by Lai et al. (59) demonstrated that short-term milk production rate decreased with inter-pump intervals ≥5 h and recommended ≥5 daily pumping sessions at regular intervals with a maximum inter-pump interval of 7 h (59). Mago-Shah et al. did not report inter-pump intervals, so it is unclear whether time of day (e.g., early morning) versus inter-pump interval is the primary determinant of subsequent MOM synthesis. Certainly, longer inter-pump intervals contribute to decreased MOM synthesis via the autocrine/paracrine regulatory pathways and the lack of frequent suckling-induced prolactin secretion from the pituitary (60, 61). While we did not routinely have pumping data available on day 5 due to the enrollment time frame through day 6 for ReDiMOM (52), we found that women were unlikely to pump in the early morning after week 1.

We found dramatically different trajectories when examining daily pumping sessions and daily pumping minutes based on whether mothers who had stopped pumping were included versus excluded from analyzes. Although the number of daily pumping sessions were relatively consistent for mothers while pumping, the inclusion of mothers who stopped pumping illustrated their impact with a downward trajectory in the average number of pumping sessions and daily pumping minutes. Inclusion of mothers who *start* pumping overall versus inclusion of mothers *only while* they are pumping is an important methodological consideration in future research.

Our study has several strengths, including the detailed collection of daily pumping sessions through maternal pumping logs that minimized recall bias; our characterization of pumping patterns by WOL and by maternal race/ethnicity, insurance, age and infant GA, which have been demonstrated to be associated with MOM feeding duration during the NICU hospitalization; and our ability to investigate inter-subject versus intra-subject variation in pumping patterns over time. However, there are limitations that should also be considered. First, we relied on maternal pumping records rather than objective pumping data, however, most studies rely on self-report. ReDiMOM is collecting pumping data using a smart pump that enables electronic capture and storage of pumping behaviors for mothers in the intervention arm, so future analyses can compare the accuracy of maternal self-report with smart pump logs and milk weight data (52). Another limitation is variability in the availability of pumping data for the first week postpartum. Mothers were eligible for enrollment into ReDiMOM from prenatal hospitalization through 144 h (6 days) postpartum, therefore, pumping records may have been missing during the pre-enrollment period. Furthermore, data were not available for the time of first pumping, which could significantly influence lactation outcomes since Parker et al. showed in their randomized controlled trial that pumping initiation within the first 6 h postpartum was associated with greater MOM volume and duration in a similar population (39).

## 5 Conclusion

The data presented here provide valuable insights into pumping behaviors of a racially, ethnically, and socioeconomically diverse group of mothers of VPT infants. Taken together, the large variation in pumping behaviors by race/ethnicity and socioeconomic status (payment source as proxy) may contribute to disparity in MOM provision for VPT infants, which supports our previous findings that pumping behaviors mediate differences in MOM provision (35). The reasons underlying these disparities in pumping behaviors are unknown and likely multifactorial, with potential reasons including racial/ethnic differences in return-to-work timing, unpaid workload, lactation-supportive work conditions, maternal health conditions, cultural and familial support for MOM provision, and implicit and explicit biases that may impact mothers in the NICU (33, 35, 47, 62–70). Measuring and acknowledging variation in pumping is a first step (71), which highlights the need to further understand barriers to pumping in order to develop appropriate interventions, education and quality improvement efforts.

## Data availability statement

The raw data supporting the conclusions of this article will be made available by the authors upon completion of the randomized controlled trial.

## Ethics statement

The studies involving humans were approved by Rush University Institutional Review Board. The studies were conducted in accordance with the local legislation and institutional requirements. Written informed consent for participation in this study was provided by the participants’ legal guardians/next of kin.

## Author contributions

AP: Conceptualization, Funding acquisition, Investigation, Methodology, Writing – original draft, Writing – review & editing. AT: Data curation, Writing – review & editing. AB: Data curation, Investigation, Writing – review & editing. JJ: Data curation, Investigation, Writing – review & editing. KM: Data curation, Investigation, Writing – review & editing. DM: Data curation, Investigation, Writing – review & editing. PM: Conceptualization, Investigation, Writing – review & editing. TJ: Conceptualization, Formal analysis, Funding acquisition, Investigation, Methodology, Visualization, Writing – original draft, Writing – review & editing.
