# Supplementary material for: Where does the time go? Temporal patterns of pumping behaviors in mothers of very preterm infants vary by sociodemographic and clinical factors
Source: Front Nutr. 2024 Jan 30;11:1278818. doi: 10.3389/fnut.2024.1278818 (PMC10861725; doi:10.3389/fnut.2024.1278818)
Supplement: Supplementary file 1 [file Data_Sheet_1.PDF]

Supplemental Figure 1. Distribution of Mothers by Proportion of Neonatal Intensive Care Unit Days with No Pumping Sessions

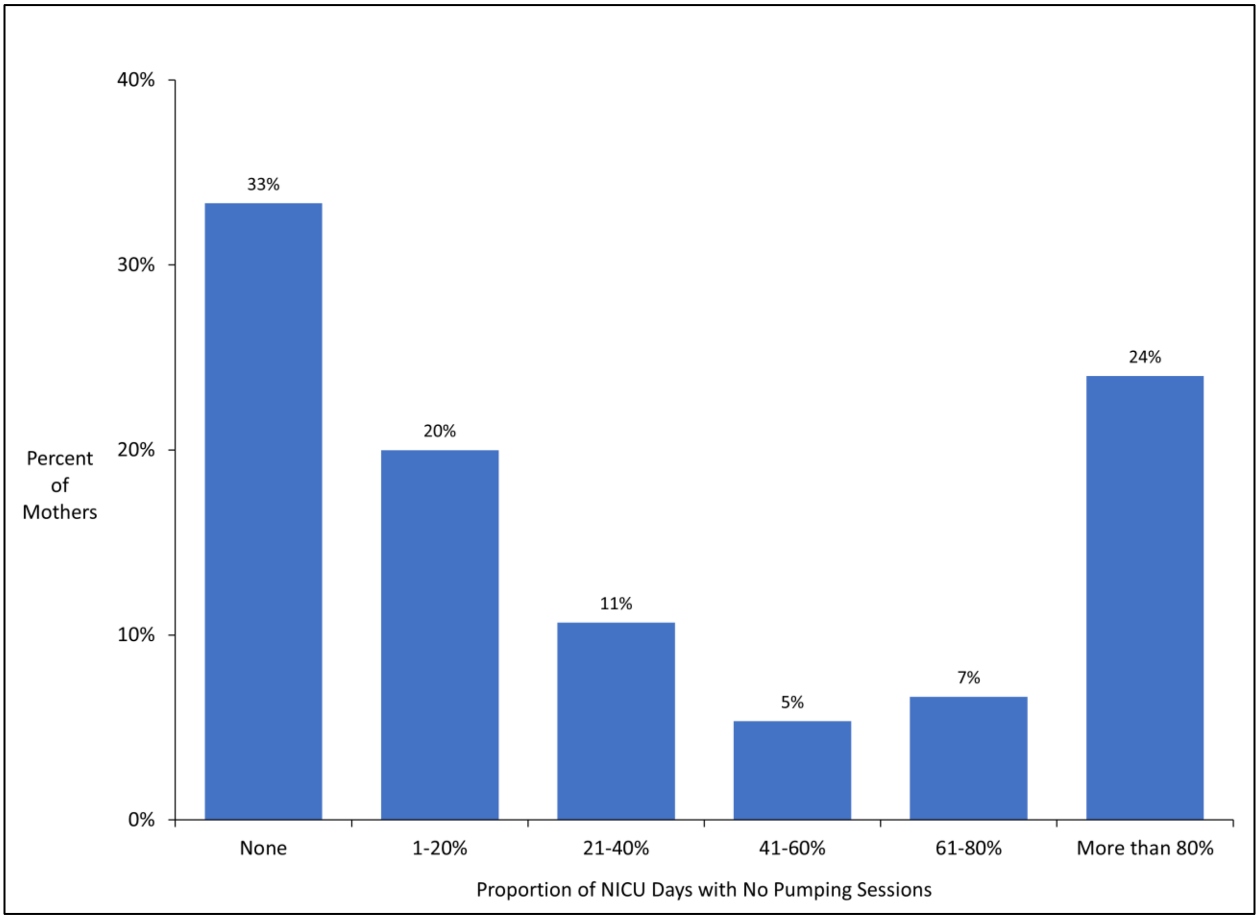

Note: Number of neonatal intensive care unit (NICU) days in denominator excludes days prior to date of first pumping session.
