# Supplementary material for: Where does the time go? Temporal patterns of pumping behaviors in mothers of very preterm infants vary by sociodemographic and clinical factors
Source: Front Nutr. 2024 Jan 30;11:1278818. doi: 10.3389/fnut.2024.1278818 (PMC10861725; doi:10.3389/fnut.2024.1278818)
Supplement: Supplementary file 2 [file Data_Sheet_2.PDF]

Supplemental Figure 2. Heat Maps of Daily Pumping Sessions, Daily Pumping Minutes and Pumping Patterns

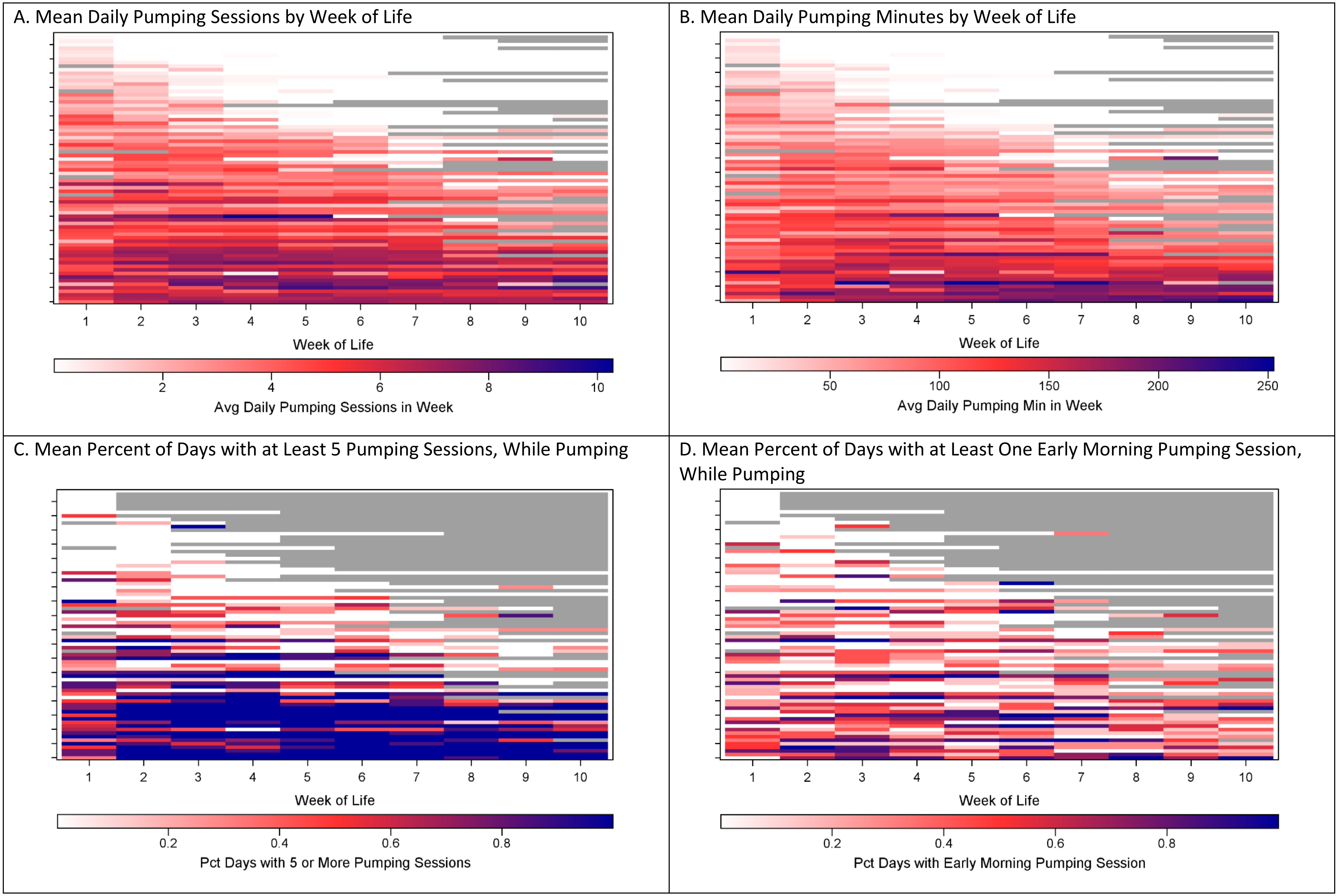

Note: Grey shading indicates weeks after discharge (mean daily pumping sessions, mean daily pumping minutes) or weeks after last pumping date (mean percent of days with at least 5 pumping sessions, mean percent of days with at least one early morning pumping session)
