# Supplementary material for: Where does the time go? Temporal patterns of pumping behaviors in mothers of very preterm infants vary by sociodemographic and clinical factors
Source: Front Nutr. 2024 Jan 30;11:1278818. doi: 10.3389/fnut.2024.1278818 (PMC10861725; doi:10.3389/fnut.2024.1278818)
Supplement: Supplementary file 3 [file Table_1.DOCX]

Supplemental Table 1

Between-Subject versus Within-Subject Variation in Outcomes

|  | Mean | Overall SD | Between-Subjects SD | Within-Subjects SD |
| --- | --- | --- | --- | --- |
| Average Daily Pumping Sessions in Week | 3.16 | 2.71 | 2.43 | 1.14 |
| Average Daily Pumping Minutes in Week | 69.78 | 63.15 | 56.19 | 27.67 |
| Average Percent of Days with at Least 5 Pumping Sessions in Week, While Pumping | 46.15 | 42.26 | 37.93 | 17.70 |
| Average Percent of Days with at Least One Early Morning Pumping Session in Week, While Pumping | 27.70 | 30.22 | 22.47 | 20.61 |
